# Diabetes-induced oxidative stress mediates upregulation of RhoA/Rho kinase pathway and hypercontractility of gastric smooth muscle

**DOI:** 10.1371/journal.pone.0178574

**Published:** 2017-07-05

**Authors:** Sunila Mahavadi, Wimolpak Sriwai, Olivia Manion, John R. Grider, Karnam S. Murthy

**Affiliations:** Department of Physiology and Biophysics, VCU Program in Enteric Neuromuscular Sciences, Virginia Commonwealth University, Richmond, Virginia, United States of America; University of Texas Medical Branch, UNITED STATES

## Abstract

The pathogenesis of diabetes-associated motility disorders are multifactorial and attributed to abnormalities in extrinsic and intrinsic innervation, and a decrease in the number of interstitial cells of Cajal, and nNOS expression and activity. Here we studied the effect of hyperglycemia on smooth muscle function. Using smooth muscles from the fundus of ob/ob mice and of wild type (WT) mice treated with 30 mM glucose (HG), we identified the molecular mechanism by which hyperglycemia upregulates RhoA/Rho kinase pathway and muscle contraction. RhoA expression, Rho kinase activity and muscle contraction were increased, while miR-133a expression was decreased in smooth muscle of ob/ob mice and in smooth muscle treated with HG. Intraperitoneal injections of pre-miR-133a decreased RhoA expression in WT mice and reversed the increase in RhoA expression in ob/ob mice. Intraperitoneal injections of antagomiR-133a increased RhoA expression in WT mice and augmented the increase in RhoA expression in ob/ob mice. The effect of pre-miR-133a or antagomiR-133a *in vitro* in smooth muscle treated with HG was similar to that obtained *in vivo*, suggesting that the expression of RhoA is negatively regulated by miR-133a and a decrease in miR-133a expression in diabetes causes an increase in RhoA expression. Oxidative stress (levels of reactive oxygen species and hydrogen peroxide, and expression of superoxide dismutase 1 and NADPH oxidase 4) was increased in smooth muscle of ob/ob mice and in HG-treated smooth muscle. Treatment of ob/ob mice with N-acetylcysteine (NAC) *in vivo* or addition of NAC *in vitro* to HG-treated smooth muscle reversed the effect of glucose on the expression of miR-133a and RhoA, Rho kinase activity and muscle contraction. NAC treatment also reversed the decrease in gastric emptying in ob/ob mice. We conclude that oxidative stress in diabetes causes a decrease in miR-133a expression leading to an increase in RhoA/Rho kinase pathway and muscle contraction.

## Introduction

Diabetes Mellitus is associated various gastrointestinal complications including delayed gastric emptying, constipation, diarrhea and fecal incontinence [1–3]. Several previous investigations have examined the role of oxidative stress in the development of diabetes-mediated disorders and considered reactive oxygen species (ROS), such as superoxide, a causal link between hyperglycemia and other metabolic abnormalities in the development of many complications of diabetes [4–10]. ROS can be generated within living cells by several sources; mitochondria, plasma membrane NADPH oxidase (NOX) and different enzymes involved in redox reactions such as several oxidases, peroxidase, cytochromes, mono and di-oxygenases and uncoupled NOS [11–14]. Physiological ROS levels play an important role in intracellular signaling as second messengers; however, when ROS production is exacerbated or scavenging is insufficient, dysregulation of many biological processes occurs [15].

Altered gastrointestinal motility in diabetes has been correlated with the changes in both autonomic and enteric nervous system and abundance of interstitial cells of Cajal (ICC) [16–27]. The role of altered intrinsic signaling pathways that lead to smooth muscle contraction in motility disorders is largely unknown. Smooth muscle contraction is regulated by phosphorylation of Ser^19^ on the 20 kDa regulatory chain of myosin II (MLC_20_) by two enzymes known as myosin light chain kinase (MLCK) and myosin light chain phosphatase (MLCP) [28–36]. An increase in intracellular cytosolic Ca^2+^, either through Ca^2+^ influx and/or release from intracellular stores, by contractile agonists leads to Ca^2+^/CaM-dependent activation of MLCK which is essential for activation of actin-activated myosin ATPase, interaction of actin and myosin, and smooth muscle contraction. In addition, G protein-coupled receptor agonists also evoke Ca^2+^-independent contractions that are mediated by two RhoA-dependent pathways. One pathway involves phosphorylation of the regulatory subunit of MLCP by Rho kinase and the other pathway involves phosphorylation of CPI-17 by protein kinase C (PKC), resulting in inhibition of MLCP activity and increase in MLC_20_ phosphorylation [28,29,31,32].

MicroRNA (miRNA) profiling has been performed in numerous types of tissues exposed to oxidative stress, suggesting the importance of miRNA modulation in the cellular response to redox imbalance [37–41]. The miR-133 family of miRNAs is the most highly expressed miRNAs in cardiac myocytes [42]. The RNA hybrid analyses of human and mouse mRNAs for RhoA revealed putative binding sites of miR-133a in their 3’UTR [43]. RhoA protein expression is negatively regulated by miR-133a in bronchial smooth muscle and cardiomyocytes [42, 44, 45]. In the present study, we tested the hypothesis that down-regulation of miRNA-133a due to oxidative stress mediates up-regulation of RhoA/Rho kinase pathway leading to hypercontractility and delayed gastric emptying in diabetes.

## Materials and methods

### Materials

RNAqueous^™^ kit, MIRVANA miRNA Kit, High Capacity cDNA RT Kit, miR-RT Kit, miRNA Assay mix Kit, miR-133 precursor and antagomir, and RAGE (receptor for advanced glycation end products) assay mix were obtained from Thermo Fisher Scientific, Waltham, MA; OxiSelect^™^ Hydrogen Peroxide assay Kit, and OxiSelectTM ROS assay kit were from Cell Biolabs, Inc. San Diego, CA; Myelin basic protein (MBP) was from Upstate Biotechnologies, Lake Placid, NY; RAGE, RhoA and phospho-specific MYPT1 (Thr^696^) antibodies were from Santa Cruz Biotechnology Inc, Santa Cruz, CA; [γ-32p]ATP was from Perkin Elmer Life Sciences, Boston, MA; Western blot supplies were from Bio-Rad, Hercules, CA; all other supplies were from Sigma, St. Louis, MO; and Fisher Scientific, Asheville, NC.

Wild type (WT) (C57BL/6) and ob/ob mice (B6.Cg-Lep^ob/J^) were purchased from Jackson Laboratories and housed on a 12h/12h dark/light cycle and allowed food and water ad libitum in the animal facility administered by the Division of Animal Resources, Virginia Commonwealth University. All procedures were approved by the Institutional Animal Care and Use Committee of the Virginia Commonwealth University.

### Preparation of dispersed and cultured smooth muscle cells

The mice were anesthetized by CO_2_ inhalation/asphyxiation followed by cervical dislocation. The stomach was removed and gastric muscle layer was separated from the mucosa by scraping. Smooth muscle cells from the fundus muscle layer were isolated by sequential enzymatic digestion, filtration, and centrifugation as described previously [30, 31]. The muscle layer was chopped into small pieces and incubated for 30 min in a smooth muscle buffer (NaCl 120 mM, KCl 4 mM, KH_2_PO_4_ 2.6 mM, CaCl_2_ 2.0 mM, MgCl_2_ 0.6 mM, HEPES (N-2-hydroxyethylpiperazine-N’ 2-ethanesulfonic acid) 25 mM, glucose 14 mM, and essential amino acid mixture 2.1% (pH7.4)) at 31°C containing 0.1% collagenase (300 U/ml) and 0.01% soybean trypsin inhibitor (w/v). The partly digested tissues were washed twice with 50 ml of collagenase-free smooth muscle buffer and the muscle cells were allowed to disperse spontaneously for 30 min. Cells were harvested by filtration through 500 μm Nitex and centrifuged twice at 350 *g* for 10 min to eliminate broken cells and organelles.

Dispersed muscle cells were resuspended in DMEM containing penicillin (200 U/ml), streptomycin (200 μg/ml), gentamycin (100 μg/ml), amphotericin B (2.5 μg/ml) and 10% fetal bovine serum (DMEM-10). The muscle cells were plated at a concentration of 1x10^5^ cells/ml and incubated at 37°C in a CO_2_ incubator. DMEM-10 medium was replaced every three days for 2–3 weeks until confluence was attained. All experiments were done on cells in the first passage.

### Isolation of RNA and quantitative PCR

Total RNA was isolated from gastric smooth muscle from WT and ob/ob mice with TRIzol^®^ reagent and from cultured muscle cells treated with 5.5 mM (normal glucose, NG) and 30 mM glucose (high glucose, HG) for 48 h using Ambion RNA isolation kit and then treated with TURBO DNase. RNA from each preparation was reversely transcribed using the High-Capacity cDNA Reverse Transcription kit in a 20-μl reaction volume. Quantitative RT-PCR (qRT-PCR) was performed on cDNA samples using specific primers designed from known sequences in mouse using SYBRgreen or Taqman PCR Mastermix. The target gene copy number is quantified by measuring threshold cycle parameter, defined as the fractional cycle at which the fluorescence generated by cleavage of probe passes a fixed threshold above the baseline, and by using a standard curve to determine the starting copy number. The primers are designed to satisfy the requirements for use of the 2^−ΔΔCt^ quantification method and normalize to GAPDH expression. Final results are expressed as fold difference in expression in samples from ob/ob mice relative to samples from WT, and HG-treated samples relative to NG-treated samples. All PCR reactions were performed in an ABI stepOne Plus PCR. Specific primers for RhoA, superoxide dismutase (SOD-1) and NOX-4 were designed based on the mouse cDNA sequence. RhoA: Forward 5’TGAAGCAGGAGCCGGTAAA3’ Reverse: 5’ CAAAAGCGCCAATCCTGTTT 3’; SOD-1: Forward: 5’ CCCGGCGGATGAAGAGA 3’ Reverse: 5’ ACCGTCCTTTCCAGCAGTCA 3’. NOX-4: Forward: 5’ CTG GGT GTG CAG AGA CAT CC 3’ Reverse: 5’ CCG AGG ACG CCC AAT GAA AA 3’. β-actin: Forward: 5’ CGT GCG TGA CAT CAA AGA GAA 3’ Reverse: 5’ GGC CAT CTC CTG CTC GAA 3’.

### miR-133a and miR-25 expression by qRT-PCR

The expression levels of miR-133a and miR-25 were detected by qRT-PCR assay. Briefly, miRNA was isolated from the smooth muscle of WT and ob/ob mice and muscle strips treated with HG for 48 h using the MIRVANA kit according to the manufacturer's instructions. This protocol allows the isolation of total RNA enriched with miRNAs. RNA concentration and quality were determined by Nanodrop. Then, miRNA expression levels were quantitated using a microRNA assay kit according to the manufacturer’s protocol. The two-step protocol involves reverse transcription with a miRNA-specific primer to convert miRNA to complementary DNA, followed by qRT-PCR with TaqMan probes. The universal small nucleolar RNA 202 and 234 (snoRNA202 and snoRNA234) was used as an endogenous control for miRNAs. Each sample was examined in triplicate. Final results are expressed as fold difference in expression in ob/ob mice relative to WT mice, and HG-treated cells relative to NG-treated cells.

### Induction of antagomir miR-133a (antagmiR-133a) and precursor miR-133a (pre-miR-133a)

Six-week-old WT and ob/ob mice were deprived of food and water for 24 h prior to the experiment. Following food and water deprivation, the body weights were measured and then randomly assigned to 3 groups (3 WT mice and 3 ob/ob mice for PBS; 3 WT mice and ob/ob mice for antagomiR-133a; 3 WT mice and ob/ob mice for pre-miR-133a). Pre-miR-133a and antagomiR-133a were dissolved in saline as the final concentration is 10 μg/μl and respective groups of mice were given consecutive doses of 5 mg/kg of body weight over 3 days or an equal volume of saline to control mice. Animals were fed for 1 week. After one-week, mice were sacrificed and the stomachs were collected.

### Transfection of pre-miR-133a or antagomiR-133a in cultured smooth muscle cells

Confluent smooth muscle cells in the first passage on six-well plates were transiently transfected with the pre-miR-133a or antagomiR-133a using Lipofectamine 2000 according to the manufacturer's instructions. Briefly, 10 nM pre-miR-133a vector in 125 μl Opti-MEM medium were mixed with 5 μl Lipofectamine 2000 in 125 μl Opti-MEM. The mixture was incubated at room temperature for 20 min and added to wells containing 1.5 ml DMEM with 10% FBS for 1 day. The medium was then replaced with DMEM with 10% FBS plus antibiotics for 2 days. Cells were maintained for a final 24 h in DMEM without FBS before experiments were started. Transfection efficiency was monitored by cotransfection of pSIREN-DNR-DsRed. Analysis by fluorescence microscopy showed that ~75% of the cells were transfected [30, 32].

### Western blot analysis

Expression of RhoA, NOX-4 and RAGE was measured using specific antibodies and phosphorylation of MYPT1 was measured using phospho-specific antibody. Muscle strips from WT and ob/ob mice and control muscle strips treated with HG for 48 h were solubilized in Triton X-100-based lysis buffer plus protease and phosphatase inhibitors (100 μg/ml PMSF, 10 μg/ml aprotinin, 10 μg/ml leupeptin, 30 mM sodium fluoride and 3 mM sodium vanadate). After centrifugation of the lysates at 20000 *g* for 10 min at 4°C, the protein concentrations of the supernatant were determined with a Dc protein assay kit from Bio-Rad. An equal amount of proteins were fractionated by SDS/PAGE and transferred on to PVDF membrane. Blots were blocked in 5% (w/v) non-fat dried milk/TBS-T [Tris-buffered saline (pH 7.6) plus 0.1% Tween-20] for 1 h and then incubated overnight at 4°C with RhoA, NOX-4 or RAGE antibodies or phospho-specific antibody to MYPT1(Thr^696^) (1:1000) in TBS-T plus 1% (w/v) non-fat dried milk. After incubation for 1 h with the horseradish-peroxidase-conjugated corresponding secondary antibody (1:2000) in TBS-T plus 1% (w/v) non-fat dried milk, immunoreactive proteins were visualized using SuperSignal Femto maximum sensitivity substrate kit (Pierce). All washing steps were performed with TBS-T. The protein bands were identified by enhanced chemiluminescence reagent [31, 32].

### Assay for Rho kinase activity

Rho kinase activity was determined by immunokinase assay in cell extracts as described previously [31]. Rho kinase immunoprecipitates were washed with phosphorylation buffer and incubated for 5 min on ice with 5 μg of myelin basic protein. Kinase assays were initiated by the addition of 10 μCi of [^32^P]ATP (3,000 Ci/mmol) and 20 μM ATP, followed by incubation for 10 min at 37°C. [^32^P]myelin basic protein was absorbed onto phosphocellulose disks, and free radioactivity was removed by repeated washings with 75 mM phosphoric acid. The amount of radioactivity on the disks was measured by liquid scintillation.

### Assay for hydrogen peroxide (H_2_O_2_)

OxiSelect^™^ Hydrogen Peroxide assay kit was used in the experiments according to the protocol provided by the company. Briefly, cells were homogenized and sonicated in PBS containing butylated hydroxytoluene. Twenty-five micro liters of the homogenates or standards were added to the microtiter plate wells followed by 250 μl of aqueous working reagent containing xylenol orange 1:100; sorbitol 1:40, and AFS reagent 1:100 to each well. The well contents were mixed thoroughly and incubated on a shaker for 30 min at room temperature. Finally, the plate was read at 540–600 nm absorbance and the concentration of H_2_O_2_ within samples was calculated by comparing the sample absorbance to the standard curve.

### ROS quantification

OxiSelectTM ROS assay kit was used in the experiments according to the protocol provided by the company. Briefly, muscle cells were placed in a clear 96-well cell culture plate overnight in the incubator. 2,7-dichlorofluorescein diacetate/media solution was added to the cells, which then exposed to HG for 48 h. Finally, the treated cells were lysed by adding 100 μl of cell lysis buffer, were mixed thoroughly and incubated for 5 min at room temperature. 150 μl of the mixture was transferred to each well of a 96-well plate that is suitable for fluorescence measurement. Fluorescence was read with a fluorometric plate reader at 480/530 nm.

### Assay for myeloperoxidase (MPO)

EnzChek Myeloperoxidase activity assay kit was used in the experiments according to the manufacturer’s instructions. Briefly, gastric muscle strips from WT and ob/ob mice were homogenized in normal saline. Fifty micro liter of the homogenates or standards were added to the wells of a 96-well microplate and 50 μl of Amplex UltraRed reagent working solution was added to all sample and standard wells. The plate was incubated at room temperature for 30 min and the reaction stopped by adding 10 μl of peroxidation inhibitor. Finally, the fluorescence intensity of each sample was measured using excitation at 530 nm and emission at 590 nm and the concentration of myeloperoxidase activity of the experimental samples was measured from the standard curve.

### Contraction of muscle strips

Muscle strips from the fundus of the stomach from WT and ob/ob mice were collected and rinsed immediately in Kreb’s solution containing 118 mM NaCl, 4.8 mM KCl, 1mM MgSO_4_, 1.15 mM NaH_2_PO_4_, 15 mM NaHCO_3_, 10.5 mM glucose and 2.5 mM CaCl_2_. Also, muscle strips from WT mice were treated with NG and HG for 48 hr. The muscle strip was tied at each end with silk thread and mounted vertically in 5 ml tissue bath containing oxygenated (95% O_2_/5%CO_2_) Kreb’s solution at a pH of 7.4 at 37°C. The tissues were mounted between glass rod and isometric transducers (Grass Technologies) connected to a computer recording system (Polyview). Preparations were allowed to equilibrate for 1h at resting tension (1g) before initiation of experiments and bath buffer solution was changed every 15 minutes during equilibration. To measure the contraction, strips were treated with 10 μM acetylcholine (ACh). At the end of each experiment, the strips were blotted dry and weighed (tissue wet weight).

### Gastric emptying

WT and ob/ob mice were deprived of food and water for 24 h prior to the experiment. After 24 h of food and water deprivation, the body weights were measured and a calculated amount of food and water was supplied. After 3 h of feeding, food and water were immediately removed and the animals were allowed to starve for 4 h. After that, the body weights were measured and the mice were euthanized. The stomach was weighed with and without the contents to measure the amount of food ingested by each animal. Food retained in the stomach after 4 h was measured and expressed as milligrams per gram of body weight.

#### Statistical analysis

The results are expressed as means±SE of n experiments and analyzed for statistical significance by student’s t-test for paired and unpaired values. Differences among groups are tested by ANOVA and checked for significance via Fisher’s protected least significant difference test. P<0.05 was considered as significant.

## Results

### Upregulation of RhoA/Rho kinase pathway in diabetes

**Fig 1:**
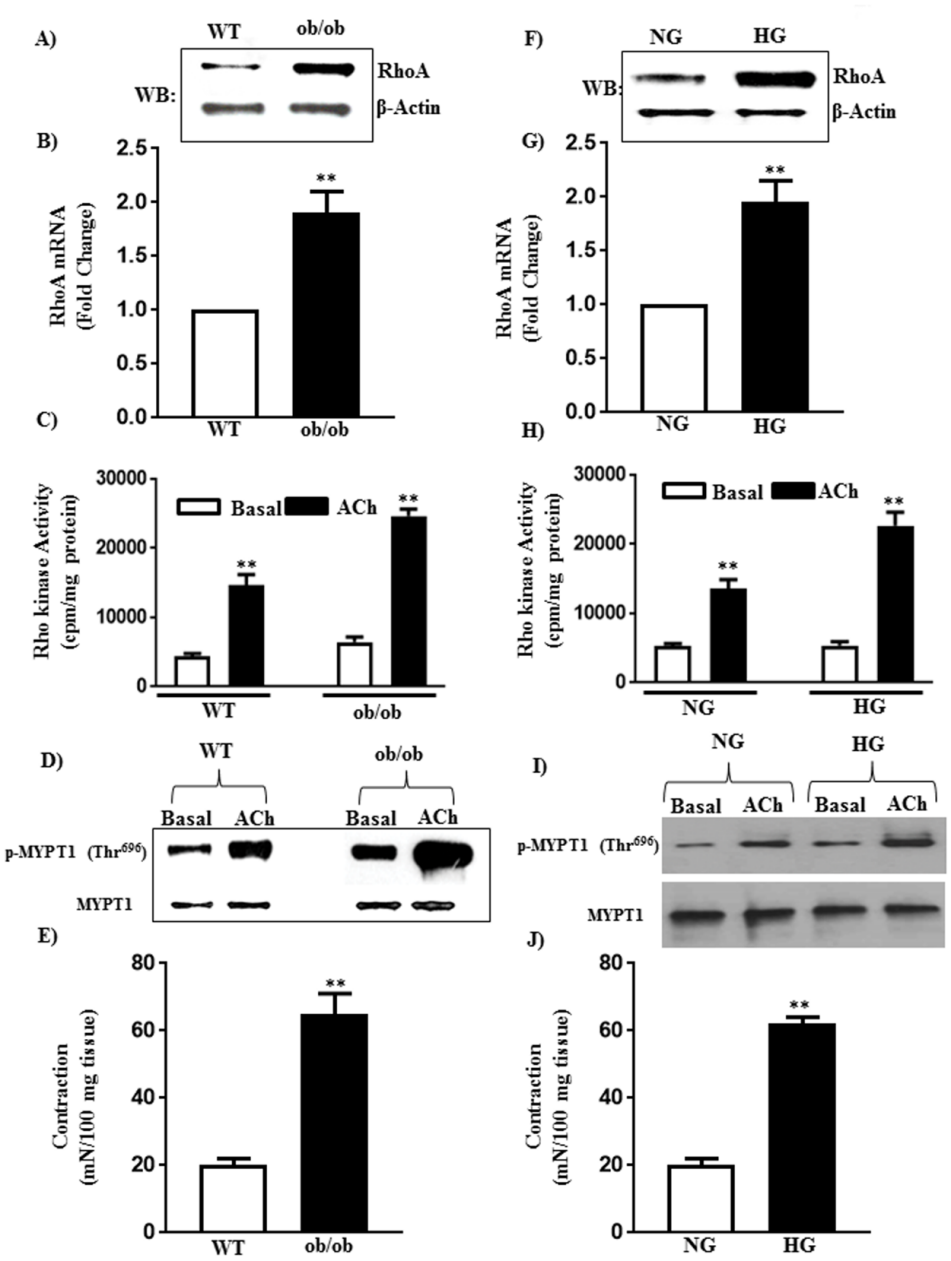
Expression of RhoA, Rho kinase activity and muscle contraction in diabetes and hyperglycemia. Smooth muscle of the fundus of wild type (WT) and ob/ob mice and smooth muscle from WT mice treated with 5.5 mM (NG) or 30 mM glucose (HG) for 48 h were used to measure the expression of RhoA, Rho kinase activity, MYPT1 phosphorylation and muscle contraction. (A, B, F, G) Expression of RhoA was measured by qRT-PCR and western blot and results with mRNA was expressed as fold change. The molecular weight of RhoA is 24 kDa. Values are means±SE of 4–6 experiments. (C and H) Rho kinase activity was measured in response to acetylcholine (ACh, 1μM) by immunokinase assay and the results are expressed as cpm/mg protein. Values are means±SE of 4–6 experiments. (D and I) MYPT1 phosphorylation was measured by western blot using phospho-specific antibody. The molecular weight of MYPT1 is 140 kDa. (E and J) Muscle contraction was measured as an increase in sustained contraction in response to acetylcholine (10 μM) in organ bath experiments and the results are expressed as mN/100 mg of tissue. Values are means±SE of 4–6 experiments.

RhoA plays an important role in the regulation of MLC_20_ phosphorylation and sustained muscle contraction [28]. Expression of RhoA mRNA was ~ 2-fold higher in the gastric smooth muscle of ob/ob mice compared to WT mice. A similar increase in RhoA protein was also obtained in smooth muscle from ob/ob mice (~3 fold increase) (Fig 1A and 1B). ACh (1 μM)-induced Rho kinase activity and Rho kinase-mediated phosphorylation of MYPT1 at Thr^696^ were also augmented (69±8% and 96±10% increase in Rho kinase activity and MYPT1 phosphorylation, respectively) in smooth muscle of ob/ob mice compared to WT mice (Fig 1C and 1D).

Similar increases in RhoA expression and Rho kinase activity in response to hyperglycemia *in vitro* were observed in muscle cells from WT mice treated with HG for 48 h. Expression, (both mRNA, and protein) of RhoA was increased (~ 3-fold increase) and ACh-induced Rho kinase activity and MYPT1 phosphorylation were augmented (67±7% and 93±10% increase) in smooth muscle cells treated with HG compared to muscle cells treated with NG (Fig 1F–1I).

Consistent with the upregulation of RhoA/Rho kinase, sustained contraction in response to 1 μM ACh was significantly higher in muscle strips isolated from ob/ob mice (225±25% increase) compared to muscle strips from WT mice. Contraction was higher in muscle strips from WT mice treated with HG for 48 h (210±20% increase) compared to muscle strips treated with NG (Fig 1E and 1J).

### Upregulation of RhoA expression by miR-133a in diabetes

Previous studies showed the expression of miR-133a in smooth muscle and binding sites for miR-133a in the 3’UTR of RhoA mRNA [43]. Binding of miRNA generally decreases the expression of proteins either due to degradation of mRNA and/or due to repression of translation [39–41]. We tested the hypothesis that the upregulation of RhoA/Rho kinase pathway in diabetes is due to decrease in miR-133a expression.

**Fig 2:**
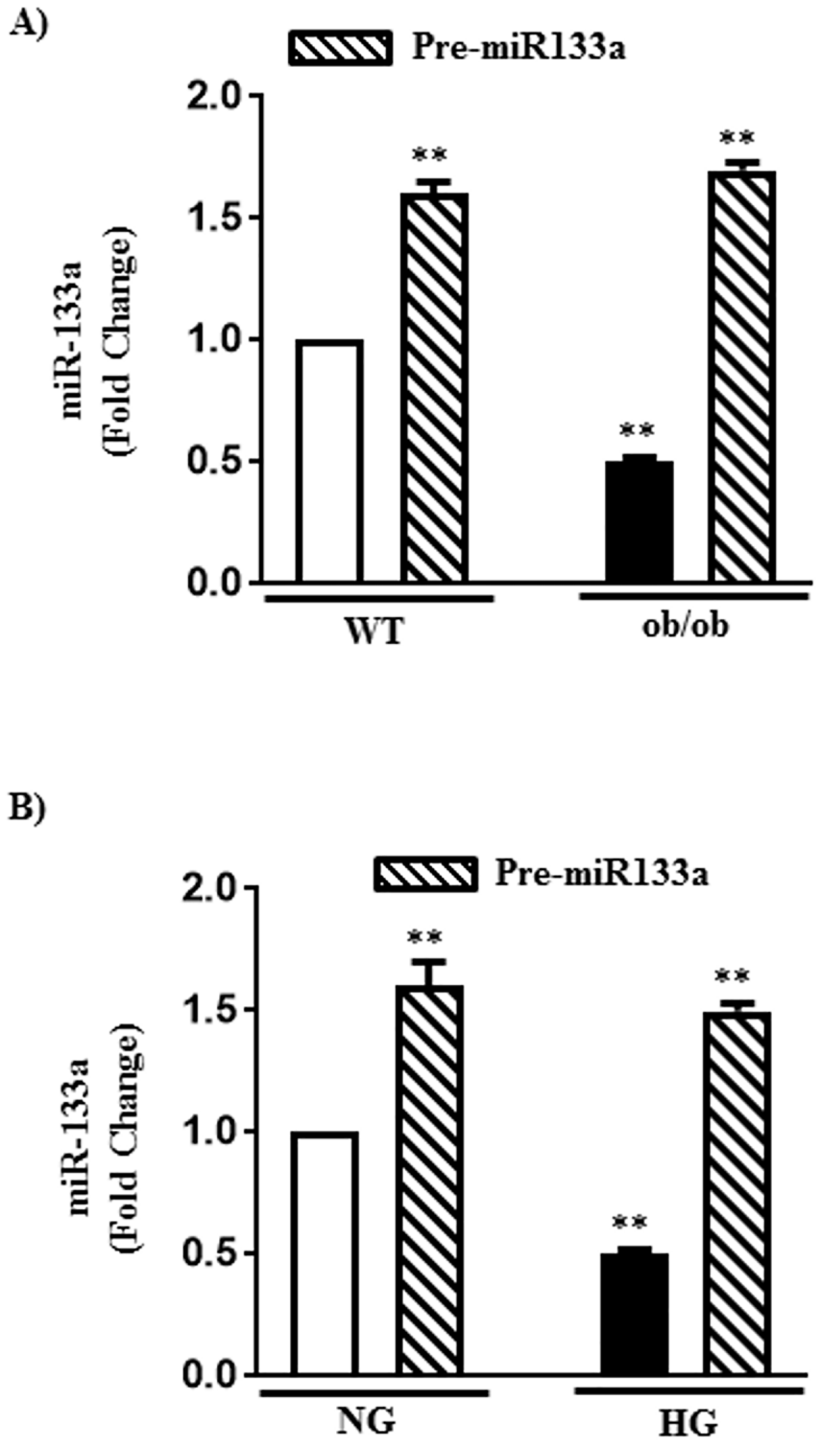
Expression of microRNA-133a in diabetes and hyperglycemia. (A) Expression of microRNA-133a (miR-133a) was measured by qRT-PCR in RNA isolated from the smooth muscle of WT or ob/ob mice with or without intraperitoneal injections of pre- miR-133a (5 mg/kg of body weight over 3 days). (B) Expression of miR-133a was measured by qRT-PCR in RNA isolated from the smooth muscle cells treated with NG or HG for 48 h in the presence or absence of transfection with pre-miR-133a. Values are means±SE of 3–5 experiments.

Expression of miRNA 133a was significantly decreased (50±8%) in the gastric smooth muscle of ob/ob mice compared to WT mice (Fig 2A). A similar decrease in miR-133a expression (50±6% decrease) was obtained in smooth muscle cells from WT mice treated with HG compared to muscle cells treated with NG (Fig 2B).

**Fig 3:**
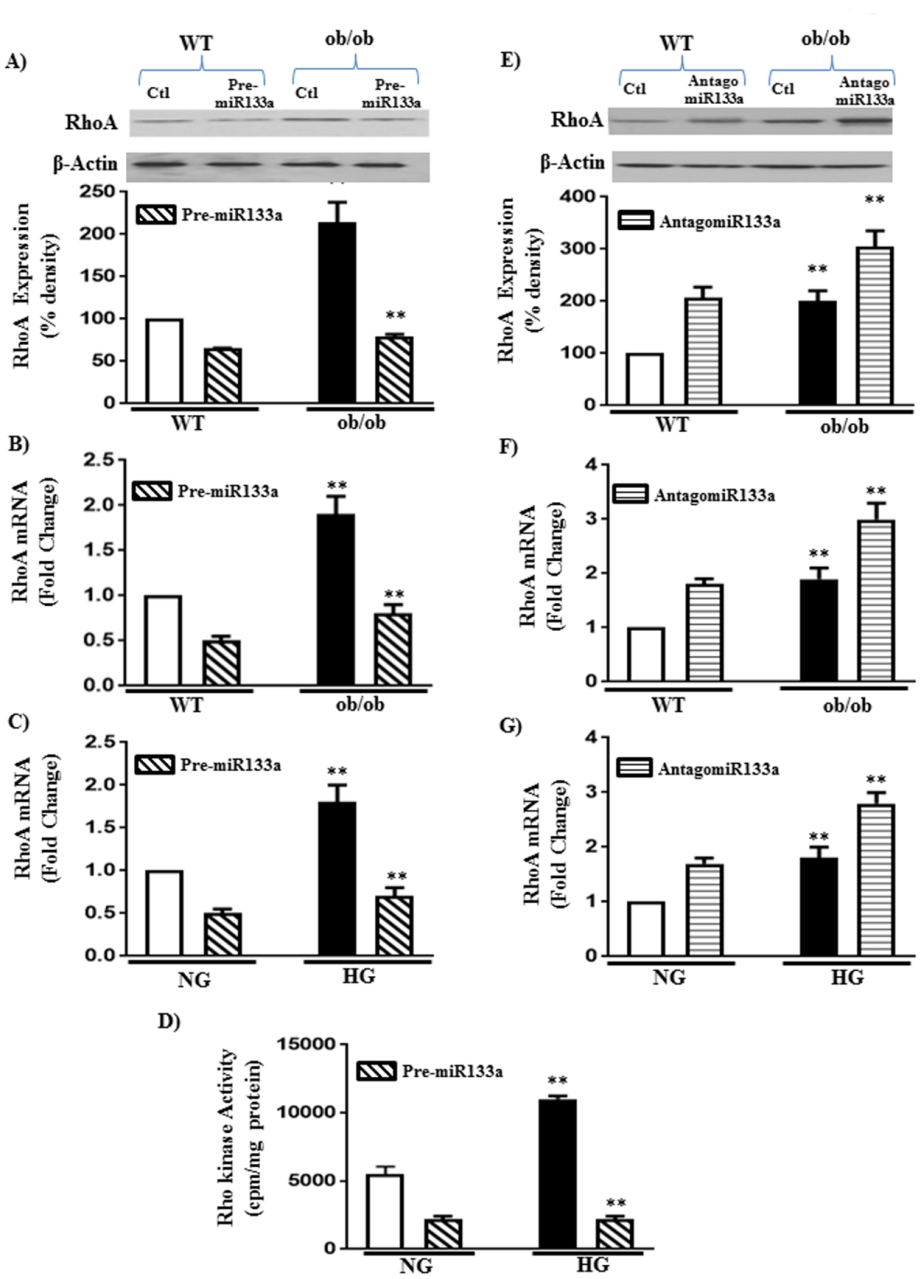
Regulation of RhoA expression and Rho kinase activity by miR-133a in diabetes and hyperglycemia. Expression of RhoA and Rho kinase activity was measured in the smooth muscle of WT or ob/ob mice with or without intraperitoneal injections pre-miR-133a or antagomiR-miR133a (5 mg/kg of body weight over 3 days) (A, B, E and F) and in smooth muscle cells treated with NG or HG for 48 h in the presence or absence of transfection with pre-miR-133a or antagomiR-133a (C and G). RhoA mRNA expression was measured by qRT-PCR and the results are expressed as fold increase from control. RhoA protein expression was measured by western blot and the results are expressed as densitometric values. The molecular weights of RhoA and β-Actin are 24 and 42 kDa, respectively. (D) Rho kinase activity was measured by immunokinase assay and the results are expressed as cpm/mg protein. Values are means±SE of 3–5 experiments.

The link between miR-133a and RhoA expression was analyzed using pre-miR-133a, which causes an increase in mature miR-133a, and antagomiR-133a, which blocks the effect of mature miR-133a, both *in vivo* and *in vitro*. Intraperitoneal (IP) injections (5 mg/kg of body weight over 3 days) of pre-miR-133a caused an increase in mature miR-133a expression in smooth muscle of WT (60±8% increase) and ob/ob (240±25% increase) mice (Fig 2A). IP injection of pre-miR-133a caused a decrease in RhoA expression in smooth muscle of WT mice and blocked the increase in RhoA expression in smooth muscle of ob/ob mice (Fig 3A and 3B).

Similarly, transfection of pre-miR-133a *in vitro* in cultured muscle cells caused an increase in mature miR-133a expression in both NG- and HG-treated cells (Fig 2B). Transfection of pre-miR-133a caused a decrease in RhoA expression in NG-treated cells and blocked the increase in RhoA expression in HG-treated cells (Fig 3C).

Consistent with the decrease in RhoA expression, transfection of pre-miR-133a attenuated ACh-induced Rho kinase activity in NG-treated smooth muscle cells (230±25% increase vs. 50±5% increase with miR-133a) and blocked the augmentation of Rho kinase activity in HG-treated cells (285±30% increase vs. 65±8% increase with miR-133a) (Fig 3D).

In contrast, IP injections of antagomiR-133a caused an increase in RhoA expression in smooth muscle from WT mice (90±10% increase) and further augmented the increase in RhoA expression in ob/ob mice (90±10% vs200±22% increase with antagmiR-133a) (Fig 3E and 3F). Similarly, transfection of antagomiR-133a *in vitro* in cultured muscle cells caused an increase in RhoA expression in NG-treated cells (80±8% increase) and further augmented the increase in RhoA expression in HG-treated cells (80±8% increase vs.180±20% increase with antagmiR-133a) (Fig 3G). These results indicate that the expression of RhoA in smooth muscle is negatively regulated by miR-133a and that decrease in miR-133a expression in both ob/ob mice and in hyperglycemia *in vitro* lead to an increase in RhoA expression and ACh-induced Rho kinase pathway.

### Increase in oxidative stress in diabetes

**Fig 4:**
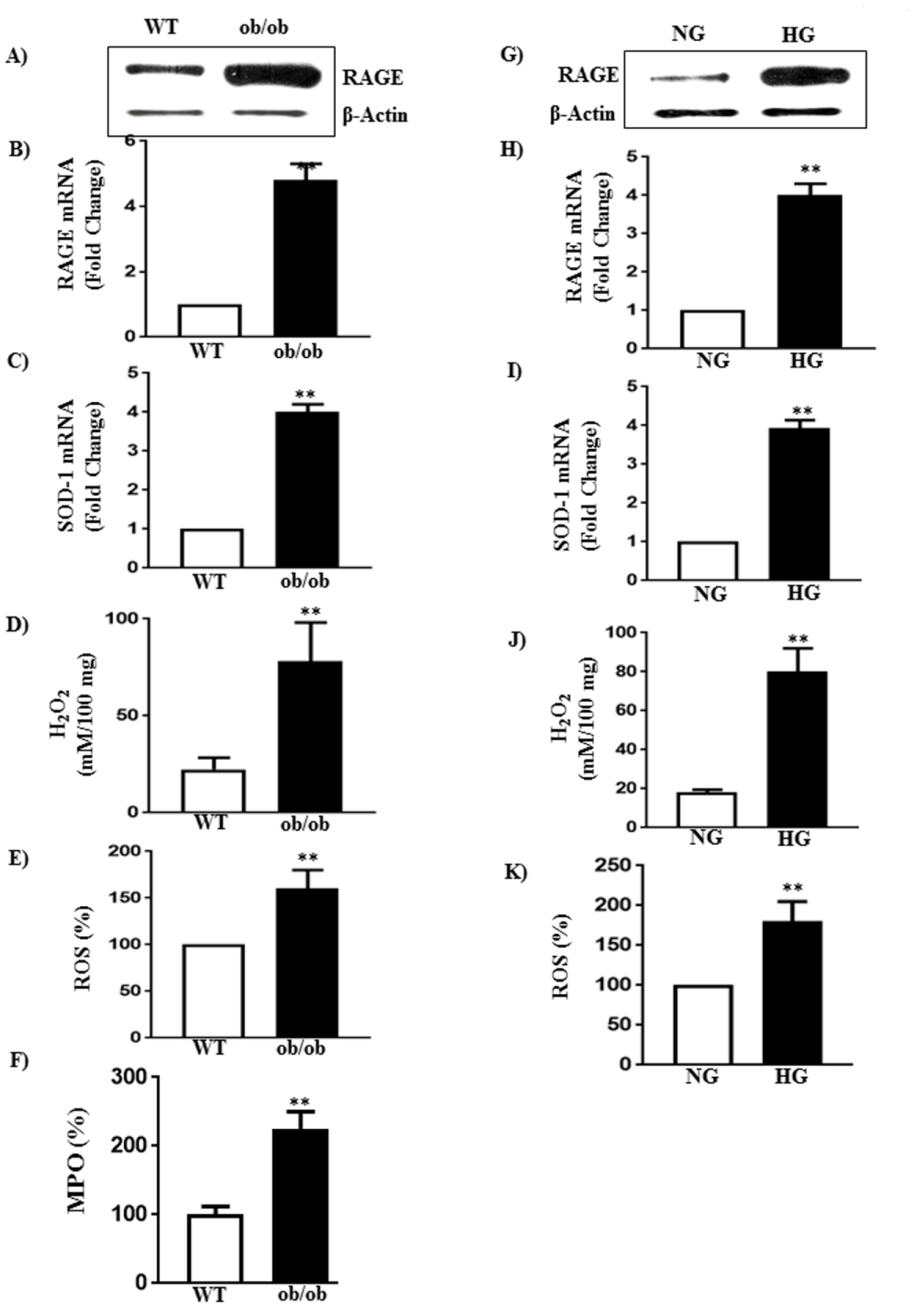
Oxidative stress in diabetes and hyperglycemia. **S**mooth muscle of the fundus of WT and ob/ob mice and smooth muscle from WT mice treated with NG or HG for 48 h were used to measure expression of the RAGE, SOD-1 and level of H_2_O_2_ and ROS. (A, B, G and H) Expression of RAGE was measured by qRT-PCR and western blot and results with mRNA was expressed as fold change. Values are means±SE of 4–6 experiments. (C and I) Expression of SOD-1 was measured by qRT-PCR and the results are expressed as fold increase from control. Values are means±SE of 4–6 experiments. (D and J) H_2_O_2_ levels were measured by calorimetry and the results are expressed as μM/100 mg. Values are means±SE of 4–6 experiments. (E and K) ROS levels were measured by fluorometry and the results are expressed as percent increase from control. Values are means±SE of 4–6 experiments. (F) MPO levels were measured by fluorometry and the results are expressed as percent increase from control. Values are means±SE of 4–5 experiments.

Increase in oxidative stress in response to diabetes was measured as an increase in the expression of RAGE and SOD-1, and formation of ROS and H_2_O_2_. Expression of RAGE mRNA and protein was ~ 3-fold higher and expression of SOD-1 mRNA was 4-fold higher in smooth muscle of ob/ob mice compared to smooth muscle of WT mice (Fig 4A–4C). The levels of ROS and H_2_O_2_ were also significantly higher (60±6% and 248±25%) in smooth muscle of ob/ob mice compared to smooth muscle of WT mice (Fig 4D and 4E). Myeloperoxidase (MPO) activity, an index of oxidative stress was also increased (125±15% increase) in smooth muscle of ob/ob mice compared to WT mice (Fig 4F). These results suggest that diabetes causes an increase in oxidative stress in the smooth muscle.

The effect of hyperglycemia on oxidative stress markers was also examined *in vitro* in smooth muscle cells of WT mice exposed to HG for 48h. Expression of RAGE mRNA and protein was 3-4-fold higher and expression of SOD-1 mRNA was 3-fold higher in muscle cells treated with HG compared to cells treated with NG (Fig 4G–4I). The levels of ROS and H_2_O_2_ (80±5%, and 344±35%) were also significantly higher in muscle cells treated HG compared to cells treated with NG (Fig 4J and 4K).

**Fig 5:**
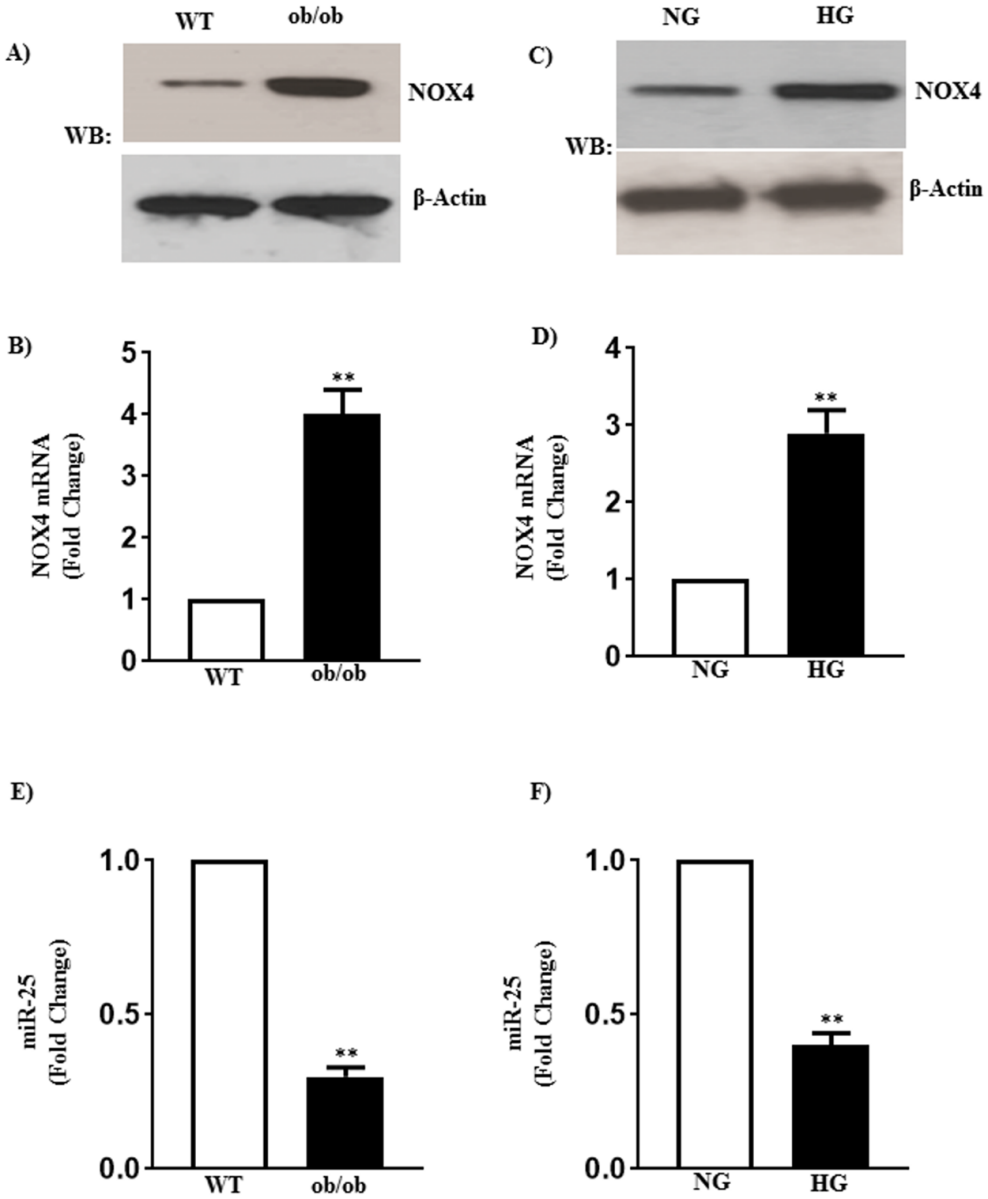
Expression of NOX-4 and microRNA-25 in diabetes and hyperglycemia. Smooth muscle of the fundus from WT and ob/ob mice, and smooth muscle of the fundus from WT mice treated with NG or HG for 48 h were used to measure the expression of NOX-4 and microRNA-25 (miR-25). (A and C) Analysis of NOX-4 expression by western blot. The molecular weights of NOX4 and β-actin are 67 and 42 kDa, respectively. (B and D) mRNA expression of NOX-4 was measured by qRT-PCR and the results are expressed as fold change. Values are means±SE of 4–6 experiments. (E and F) Expression of miR-25 was measured by qRT-PCR and results are expressed as fold change. Values are means±SE of 4–6 experiments.

Previous studies showed that an increase in oxidative stress is associated with the increase in NOX-4 expressions in vascular and airway smooth muscle [46]. As shown in Fig 5, mRNA and protein expressions of NOX-4 were increased (~ 4-fold) in smooth muscle of ob/ob mice and in smooth muscle cells from WT mice exposed to HG compared to their respective controls (Fig 5A–5D). The increase in the expression of NOX-4 expression is associated with a decrease in the expression of miR-25, a known regulator of NOX-4, (47, 48), in smooth muscle of ob/ob mice and smooth muscle of WT mice treated with HG (Fig 5E and 5F), suggesting that NOX-4 expression is negatively regulated by miR-25 and hyperglycemia-induced decrease in miR-25 expression caused an increase in NOX-4 expression. An increase in NOX-4 expression may lead to increase in oxidative stress [47,48].

### The role of oxidative stress in diabetes-induced upregulation of RhoA/Rho kinase

**Fig 6:**
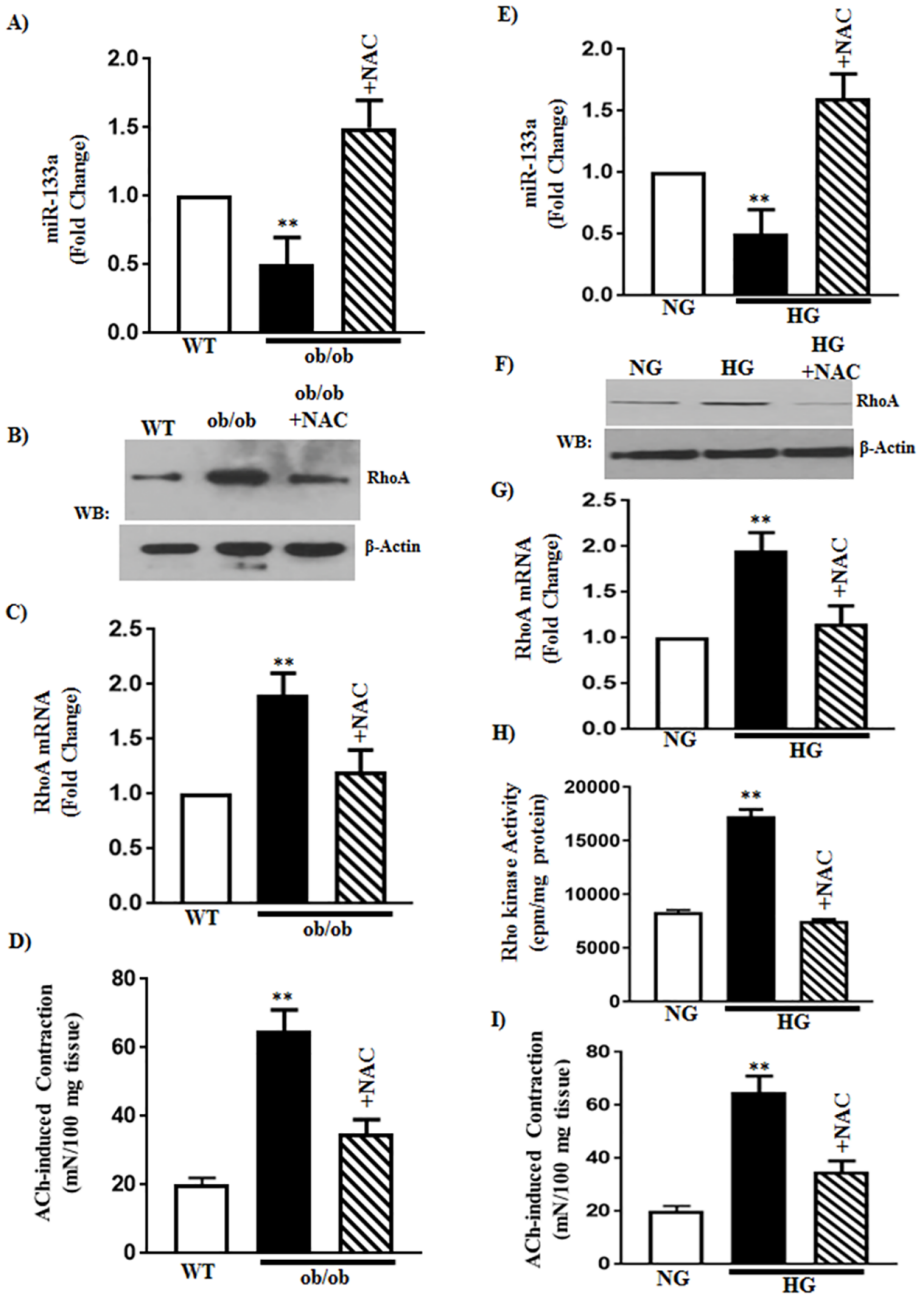
Effect of N-acetylcysteine (NAC) on the expression of miR-133 and RhoA, and muscle contraction in diabetes and hyperglycemia. (A and E) Expression of miR-133a was measured by qRT-PCR in RNA isolated from the smooth muscle of WT or ob/ob mice with or without intraperitoneal (IP) injections of NAC (300 mg/kg) and in RNA isolated from smooth muscle cells treated with NG or HG for 48 h in the presence or absence of NAC (1 mM). Results are expressed as fold change from control. Values are means±SE of 4–6 experiments. (B, C, F and G) Expression of RhoA was measured by qRT-PCR and western blot in smooth muscle of WT or ob/ob mice with or without IP injections of NAC (300 mg/kg) and in smooth muscle cells treated with NG or HG for 48 h in the presence or absence of NAC. Results are expressed as fold change from control for mRNA levels. The molecular weights of RhoA and β-actin are 24 and 42 kDa, respectively. Values are means±SE of 4–6 experiments. (H) Rho kinase activity was measured in response to acetylcholine (ACh, 1μM) by immunokinase assay and the results are expressed as cpm/mg protein. Values are means±SE of 4–5 experiments. (D and I) Muscle contraction was measured as increase in sustained contraction in response to acetylcholine (10 μM) in organ bath experiments in smooth muscle of WT or ob/ob mice with or without IP injections NAC (300 mg/kg) and in smooth muscle treated with NG or HG for 48 h in the presence or absence of NAC (1mM). Results are expressed as mN/100 mg of tissue. Values are means±SE of 4–6 experiments.

Changes in the miRNA-133a levels and miR-133a-driven RhoA expression in diabetes could be due to increase in oxidative stress. This notion was examined using the antioxidant N-acetylcysteine (NAC) both *in vivo* and *in vitro*. The effect of diabetes *in vivo* and hyperglycemia *in vitro* on the expression levels of miR-133a and RhoA and on ACh-induced Rho kinase activity and muscle contraction was blocked with NAC. Expression of miR-133a was inhibited in smooth muscle of ob/ob mice and the inhibition was reversed by treatment with NAC (300 mg/kg) for 24 h (Fig 6A). Expression of RhoA (90±10% increase) and ACh-induced muscle contraction (225±25% increase) were increased in smooth muscle of ob/ob mice and the increases in both RhoA expression (19±2% increase) and muscle contraction (75±8% increase) were reversed by treatment with NAC (Fig 6B–6D).

Similarly, expression of miR-133a was inhibited in smooth muscle of WT mice treated with HG and the inhibition was reversed by co-treatment of cells 1 mM NAC (Fig 6E). The increase in the expression of RhoA, and ACh-induced Rho kinase activity and muscle contraction in HG-treated smooth muscle were attenuated by co-treatment of cells with NAC (Fig 6F–6I). Control studies showed that the increase in H_2_O_2_ levels in response to HG was also blocked (334±35% increase vs. 67±5%) by co-treatment of cells with the NAC.

**Fig 7:**
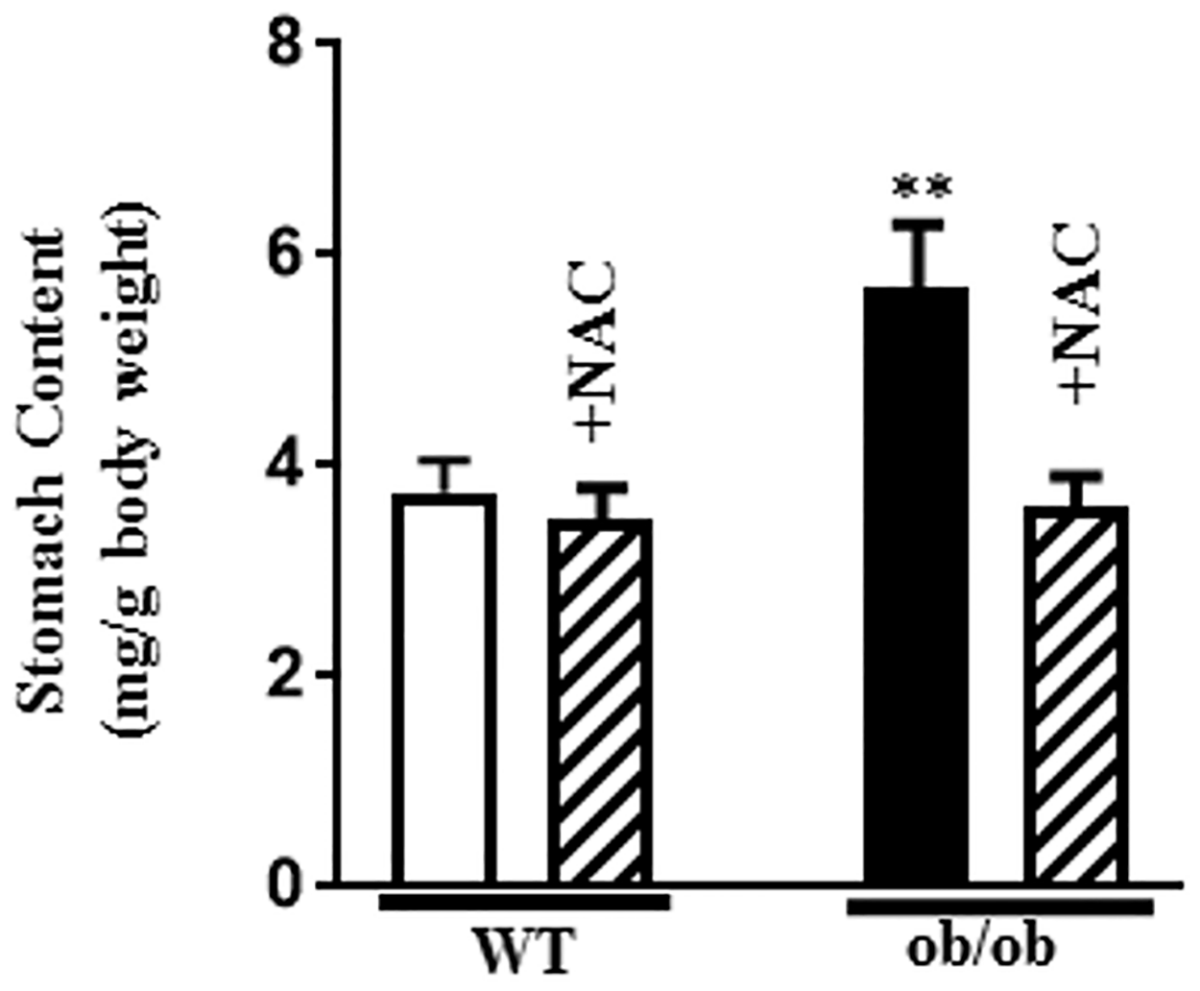
Delayed gastric emptying in diabetes. WT and ob/ob mice were treated with IP injections of NAC (300 mg/kg) and fasted for 24 h and then fed normal chow ad libitum for 4 h. Gastric emptying was monitored immediately after 3 h of feeding. Results are expressed as the amount of leftover food in the stomach as milligrams per gram of body weight. Values are means±SE of 4 experiments.

Gastric emptying was significantly delayed in ob/ob mice compared to WT mice and the delay was reversed upon treatment of ob/ob mice with NAC (Fig 7). The effect of NAC on gastric emptying in ob/ob mice is consistent with its effect on oxidative stress marker H_2_O_2_ and on the expression of miR-133a and RhoA and muscle contraction in ob/ob mice *in vivo* and in smooth muscle treated with HG *in vitro*. These results suggest that the increase in oxidative stress in diabetes *in vivo* and hyperglycemia *in vitro* causes a decrease in miR-133a expression leading to an increase in RhoA/Rho kinase pathway and muscle contraction.

## Discussion

Gastric motility is complex and requires interplay of many cell types including enteric neurons, ICC, and smooth muscle cells. The pathogenesis of the diabetes-induced motility disorders has been linked to the decrease in ICC, extrinsic and intrinsic neuropathy, and decrease in nNOS expression and activity [18–27]. Changes in the intrinsic signaling pathways that regulate smooth muscle function can also contribute to motility disorders. Others and we have shown that sustained contraction of the tonic smooth muscle is mediated by a RhoA-dependent pathway involving Rho kinase-mediated phosphorylation of MYPT1, a targeting subunit and an activator of MLCP [28,29, 49]. Phosphorylation of MYPT1 by Rho kinase leads to inhibition of MLCP activity and increase in MLC_20_ phosphorylation and muscle contraction. Several studies implicated abnormal RhoA/Rho kinase pathway in the pathophysiology of hypertension associated with diabetes [50, 51]. In the present study, we have examined the mechanisms that lead to changes in RhoA/Rho kinase pathway and contraction in response to high glucose levels using smooth muscle from the fundus of diabetic mice and smooth muscle from WT mice exposed to hyperglycemic conditions *in vitro*. Type 2 diabetic ob/ob mice were used in the present study as the obesity is closely linked to diabetes and rodent models with deficiency in leptin signaling were used as models of obesity and diabetes-related motility disorders [52, 53]. Our studies provided a link between oxidative stress, miR-133a, and RhoA in diabetes-induced hypercontraction and demonstrated that oxidative stress in response to hyperglycemia causes a decrease in miR-133a expression leading to upregulation of RhoA/Rho kinase pathway. The evidence is based on the following results: i) oxidative stress markers such as levels of ROS and H_2_O_2_ and expression of SOD-1 and RAGE were elevated in smooth muscle of ob/ob mice and in smooth muscle treated with HG; ii) expression of miR-133a was decreased, whereas expression of RhoA, and ACh-induced Rho kinase activity, MYPT1 phosphorylation and muscle contraction were increased in smooth muscle of ob/ob mice and in smooth muscle treated with HG; iii) expression of RhoA was decreased by pre-miR-133a and increased by antagmiR-133a suggesting that RhoA expression is negatively regulated by miR-133a; iv) hyperglycemia-induced increases in RhoA expression and Rho kinase activity were reversed by IP injection of pre-miR-133a in ob/ob mice *in vivo* or transfection of pre-miR-133a in culture muscle cells *in vitro*, suggesting that an increase in RhoA expression and Rho kinase activity in response to hyperglycemia was due to a decrease in miR-133a expression; and v) hyperglycemia-induced decrease in miR-133a expression, and increases in RhoA expression, Rho kinase activity and muscle contraction were also reversed by IP injections of NAC in ob/ob mice *in vivo* and by treatment of smooth muscle with HG in the presence of NAC *in vitro*, suggesting that the decrease in miR-133a expression and increase in RhoA/Rho kinase pathway and muscle contraction in response to hyperglycemia was due to an increase in oxidative stress.

The role of RhoA/Rho kinase pathway in the regulation of muscle contraction is well established in both visceral and vascular smooth muscle. Airway hyperresponsiveness in asthma and vascular hypertension in diabetes were shown to be associated with an increase in RhoA expression [43]. In line with these studies, our studies in smooth muscle from the fundus of the stomach also provide evidence for the upregulation of RhoA/Rho kinase pathway leading to hypercontraction in diabetes. In the smooth muscle of ob/ob mice and smooth muscle treated with HG *in vitro*, there was an increase in RhoA expression and ACh-induced Rho kinase activity and muscle contraction. Our studies also identified the mechanism underlying the increase in RhoA expression in diabetes. The decrease in the expression of miR-133a in diabetes causes the increase in RhoA expression. The link between miR-133a and RhoA expression was demonstrated using pre-miR-133a and antagomiR-133a both *in vivo* in ob/ob mice and *in vitro* in smooth muscle treated with HG. In both systems, the increase in RhoA expression was blocked by treatment with pre-miR-133a, whereas the increase was augmented by treatment with antagomiR-133a. These results provide evidence that in gastric smooth muscle RhoA expression is negatively regulated by miR-133a and a decrease in miR-133a expression in diabetes causes an increase in RhoA expression. Similar negative regulation of RhoA expression was reported in the smooth muscle of internal anal sphincter (IAS). The aging-induced increase in miR-133a expression was associated with a decrease in RhoA expression and IAS tone [54]. In addition to the regulation of RhoA by miR-133a, studies in human bronchial smooth muscle have identified a transcriptional regulation of RhoA expression [44, 45]. Binding sites for STAT6 and NF-κB in RhoA promoter has been identified and activation of both STAT6 and NF-κB was shown to be required for upregulation of RhoA expression in response to IL-13 in these cells [55].

PKC is also an important regulator of contraction in both vascular and visceral smooth muscle. Activation of PKC causes phosphorylation of CPI-17, an endogenous inhibitor of MLCP, and augmentation of its inhibitory effect on MLCP leading to an increase in MLC_20_ phosphorylation [28, 29, 49]. Studies by Xie et al., [56] showed an increase in the expression of both RhoA and CPI-17 in the aortic smooth muscle, but not in mesenteric artery, of db/db mice. They also showed an increase in RhoA expression and activity, and phosphorylation of CPI-17 and MYPT1 in aortic smooth muscle cells upon exposure to HG.

Smooth muscle contraction is regulated by both Ca^2+^-dependent activation of MLCK and RhoA-dependent inhibition of MLCP [28]. Increase in cytosolic Ca^2+^ either due to release via Gα_q_/PLC-β1 /IP_3_ pathway or influx via L-type Ca^2+^ channels causes activation of MLCK [28]. Studies by Descorbeth et al., [34] showed that the expression of Gα_q_, Gα_11_, PLC-β1 and PLCβ2 was upregulated in aortic smooth muscle of streptozotocin (STZ)-treated rats, and upon exposure of smooth muscle cells to HG. Studies by Pinho et al., [57] showed that the L-type Ca^2+^ channel was upregulated leading to hypercontractility in aortic smooth muscle of STZ-treated mice. The diabetes-induced hypercontractile phenotype of smooth muscle was also observed the bladder, corpus cavernosum, and uterus [58–60]. From these studies, it appears that upregulation of Ca^2+^-dependent MLCK activity and RhoA/PKC-dependent inhibition of MLCP is a general mechanism involved in smooth muscle hypercontractility in diabetes. In contrast to these studies in smooth muscle from different tissues and our studies in smooth muscle from the fundus of the stomach, studies by Touw et al., [61] showed a decreased in both cytosolic Ca^2+^ and contraction in response to KCl in distal but not proximal colon of STZ-treated mice. The decrease was attributed to iNOS-induced post-translational modification of Ca^2+^ channel. A decrease in both carbachol-and KCl-induced contraction was also reported in smooth muscle strips from the antrum of the stomach of ob/ob mice compared to control [62]. The frequencies and amplitudes of Ca^2+^ transients in response to carbachol or KCl, however, were not different between smooth muscle from ob/ob and control mice. The decrease in contraction was attributed to decrease in Rho kinase 2 expression and phosphorylation of MYPT1 [62].

Hyperglycemia-induced oxidative stress is a major risk factor in diabetes-induced complications and it is possible that the effects of oxidative stress are mediated via altered miRNA levels. Our studies also provide evidence that the decrease in miR-133a expression and increase in RhoA/Rho kinase pathway in diabetes were due to increase in oxidative stress. The effect of hyperglycemia on miR-133a expression and RhoA/Rho kinase pathway and muscle contraction was reversed by anti-oxidant NAC treatment both *in vivo* and *in vitro*. Hyperglycemia causes oxidative stress due to increased oxidation of glucose via mitochondrial electron-transport chain and due to an increase in the flux of glucose via polyol and glyceraldehyde pathways. In the polyol pathway, glucose is metabolized via sorbitol to fructose and fructose metabolites (fructose-3-P and 3-deoxyglucosone) [63–65]; the latter stimulate the formation of advanced glycation end-products (AGE) and binding of AGE to its receptor, RAGE, leading to ROS formation. In the glyceraldehyde pathway, dihydroxyacetone phosphate is metabolized to diacylglycerol (DAG), an activator of PKC isoforms (α, β, δ, ε) [46]; the latter are potent activators of membrane-associated NOX [11–14]. The glyceraldehyde pathway also yields methylglyoxal, the primary AGE precursor. Although our studies did not identify the mechanism for the decrease in miR-133a in diabetes, several studies showed that activation of RAGE by AGE causes activation of redox-sensitive transcription factors and regulation of their target genes including miRNAs [66]. The changes in miRNAs, in turn, may regulate the expression of key enzymes involved in oxidative stress (e.g., SOD1 and NOX4) [37, 47]. Downregulation of miR-25 was shown to induce NOX-4 expression in diabetic rat kidney [67]. The increase in NOX-4 expression and ROS levels observed in smooth muscle of ob/ob mice or in smooth muscle treated with HG could be due to a decrease in miR-25 expression in diabetes.

Results from our *in vivo* gastric motility demonstrating the reversal of delay in gastric emptying by NAC further demonstrates that oxidative stress in diabetes impairs smooth muscle contraction and gastric motility. Oxidative stress in diabetes appears to be the major mechanism underlying the changes in enteric neurons, ICC, and smooth muscle cells. The levels of serum malondialdehyde, a measure of oxidative stress, were significantly higher in diabetic mice with delayed gastric emptying compared to diabetic mice with normal gastric emptying [68, 69]. Heme oxygenase-1 (HO-1) in macrophages plays an important role in the protection against oxidative stress and delayed gastric emptying. Expression of HO-1 seems to be necessary to prevent the development of delayed gastric emptying [68, 69] and diabetic mice that maintain HO-1 expression did not develop delayed gastric emptying, while HO-1 induction by hemin or IL-10 reversed the delay in gastric emptying. Studies in diabetic mice also suggest that oxidative stress activates macrophages. Activation of M1 macrophages that lack HO-1 leads to the development of delayed gastric emptying. In contrast, activation of M2 macrophages protects from delayed gastric emptying [68, 69]. Thus, the effect of NAC in our studies in restoring gastric emptying could be due to its effect on multiple cell types including restoring the effect of hyperglycemia/oxidative stress on RhoA/Rho kinase pathway in smooth muscle. Several studies showed that oxidative stress is involved in the increase of contractile function of both vascular and visceral smooth muscle. For example, increase in DAG levels and PKC activity induced by high glucose in vascular smooth muscle cells was shown to be blocked by treatment with anti-oxidant α-tocopherol suggesting that the changes are due to increase in oxidative stress [70].

**Fig 8:**
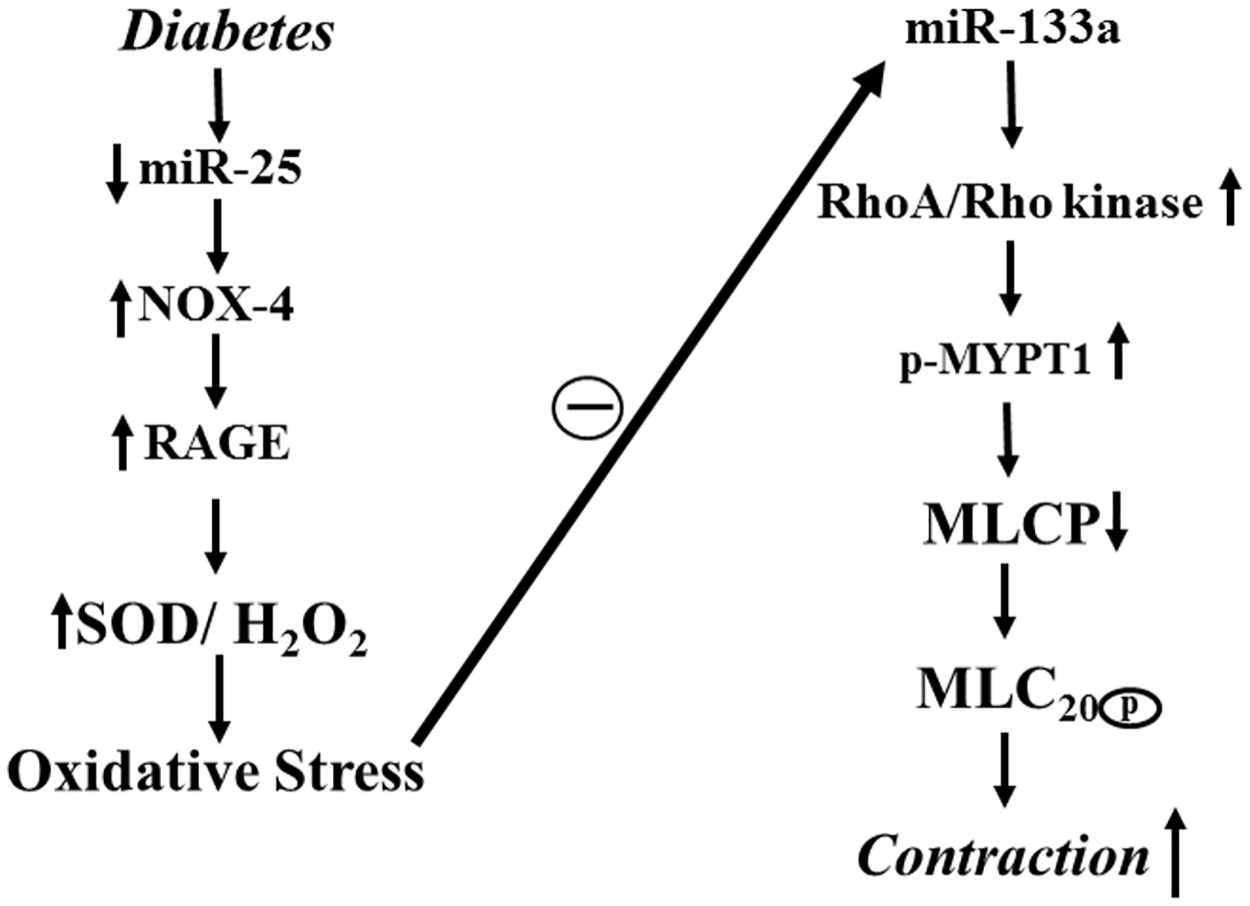
Schematic diagram of the mechanism involved in the upregulation of RhoA/Rho kinase pathway and hypercontraction in diabetes. In smooth muscle, activation of RhoA by contractile agonist causes stimulation of Rho kinase activity and Rho kinase-dependent phosphorylation of MYPT1 resulting in inhibition of MLCP activity and increase in MLC_20_ phosphorylation and muscle contraction. In diabetic smooth muscle, oxidative stress causes upregulation of RhoA/Rho kinase pathway and hypercontraction. The decrease in miR-133a expression in response to oxidative stress leads to an increase in RhoA expression, Rho kinase activity, MYPT1 phosphorylation and muscle contraction.

In conclusion, the present studies demonstrate that the expression of RhoA is negatively regulated by miR-133a and a decrease in its expression in diabetes leads to an increase in RhoA expression, agonist-induced Rho kinase activity, and muscle contraction. Our studies also suggest that oxidative stress in diabetes upregulates RhoA/Rho kinase pathway and smooth muscle contraction. An importance of oxidative stress in mediating these changes in diabetes was demonstrated using NAC which is capable of reversing the effect of diabetes on the miR-133a and RhoA expression, Rho kinase activity, muscle contraction and gastric emptying (Fig 8).
